# Trends of Increasing Medical Radiation Exposure in a Population Hospitalized for Cardiovascular Disease (1970–2009)

**DOI:** 10.1371/journal.pone.0050168

**Published:** 2012-11-28

**Authors:** Clara Carpeggiani, Patrizia Landi, Claudio Michelassi, Paolo Marraccini, Eugenio Picano

**Affiliations:** CNR, Institute of Clinical Physiology, Pisa, Italy; University of California, San Francisco, United States of America

## Abstract

**Background:**

High radiation doses employed in cardiac imaging may increase cancer frequency in exposed patients after decades. The aim of this study was to evaluate the relative trends in medical radiation exposure in a population hospitalized for cardiovascular disease.

**Methods and Results:**

An observational single-center study was conducted to examine 16,431 consecutive patients with heart disease admitted to the Italian National Research Council Institute of Clinical Physiology between January 1970 and December 2009. In all patients, the cumulative estimated effective dose was obtained from data mining of electronic records of hospital admissions, adopting the effective dose typical values of the American Heart Association 2009 statement and Mettler’s 2008 catalog of doses. Cumulative estimated effective dose per patient in the last 5 years was 22 (12–42) mSv (median, 25^th^–75^th^ percentiles), with higher values in ischemic heart disease (IHD), 37 (20–59) vs non-IHD, 13 (8–22) mSv, p<0.001. Trends in radiation exposure showed a steady increase in IHD and a flat trend in non-IHD patients, with variation from 1970–74 to 2005–2009 of +155% for IHD (p<0.001) and −1% in non-IHD (NS). The relative contribution of different imaging techniques was remodeled over time, with nuclear cardiology dominating in 1970s (23% of individual exposure) and invasive fluoroscopy in the last decade (90% of individual exposure).

**Conclusion:**

A progressive increase in cumulative estimated effective dose is observed in hospitalized IHD patients. The growing medical radiation exposure may encourage a more careful justification policy regarding ionizing imaging in cardiology patients applying the two main principles of radiation protection: appropriate justification for ordering and performing each procedure, and careful optimization of the radiation dose used during each procedure.

## Introduction

Rates of death from cardiovascular disease have been steadily declining over the past few decades, with improvements observed across most age and sex groups [1]. These improvements in prognosis of cardiovascular patients are largely due to reduction in major risk factors [2] and to the diffusion of medical diagnostic and therapeutic procedures such as coronary angiography and percutaneous coronary interventions for acute coronary syndrome (ACS) [3]. At the same time, these types of exams expose patients to ionizing radiation, which may elevate a person’s lifetime risk of developing cancer [4]. Public and governmental awareness of environmental influences on cancer risk has increased substantially in recent years. Medical imaging has been identified as one of the major causes of environmental exposure to carcinogens [5]. The collective radiation dose from medical imaging has increased by a factor of 6 in the last 2 decades [5], [6], and cardiac imaging has contributed greatly to this radiological warming [7]–[9]. Therefore, it has been recently proposed that efforts should be made to document effective total estimated effective dose per episode of care or per disease or during lifetime [10]. Total estimated effective dose can be a potential safety metric for patients with common clinical conditions [11].

The current study hypothesis was that the cumulative in-hospital radiological exposure increased significantly in adult cardiology patients over the last few decades and more so in patients with ischemic heart disease (IHD). The aim of this study was to evaluate relative trends in cumulative estimated effective dose to medical imaging radiological examinations in a population hospitalized for cardiovascular disease.

## Materials and Methods

### Ethics Statement

The study was approved by the Pisa Ethical Committee as a part (workpackage 1) of the SUIT-Heart (Stop Useless Ionizing Testing in Heart Disease) study on October 1, 2010 (Study Protocol n° 3005/2010). Written consent was given by the patients at the time of admission to the hospital for their information to be stored in the hospital database and used for research.

### Study Population

The study included 16,431 consecutive patients hospitalized at the Italian National Research Council (CNR) Institute of Clinical Physiology between January 1970 and December 2009 due to suspected or documented cardiac disease. At discharge, all demographic, history, clinical and instrumental data were collected in the Institute’s dedicated cardiovascular database [12], [13]. For this study, data on diagnosis, nuclear medicine and radiological cardiac imaging tests were considered.

### Dose Estimation

For each radiation test, the representative effective dose was estimated in milliSievert (mSv) and also with corresponding dose in multiples of chest X-rays (single postero-anterior projection, 0.02 mSv) as recommended by European Commission Medical Imaging guidelines [14], and by the UK Royal College of Radiology guidelines [15). Reference doses were taken from four sources: European [14]and UK [15] guidelines; from the American Heart Association 2009 committee [16); and when not listed elsewhere, from Mettler’s 2008 catalogue of doses of common medical imaging testing (see Table 1) [17].

**Table 1:** Representative effective doses of common cardiological examinations.

Examination	Representative effectivedose value (mSv)	Multiples of chest X-ray(PA projection)	Ref.
Chest X-ray PA	0.02	1	14,15
Chest CT	8	400	14,15
Abdomen CT	10	500	14,15
64-Slice coronary CTA without tube current modulation	15	750	16
64-Slice coronary CTA with tube current modulation	9	450	16
Diagnostic invasive coronary angiogram	7	350	16
Abdominal angiography or aortography	12	600	16
Percutaneous coronary intervention	15	750	16
Radiofrequency ablation	15	750	16
Sestamibi (1-day) stress-test MPS	9	450	16
Thallium stress-rest MPS	41	2050	16
F-18 FDG	14	700	16
Cardiac ventriculography (99mTc-labeled red blood cells)	7.8	390	17
Lung perfusion (99mTc-MAA)	2	100	17
Gallium-67 citrate	15	750	17

PA: postero-anterior. CT: computed tomography. CTA: coronary computed tomography angiography. MPS: myocardial perfusion scan. FDG: fluorodeoxyglucose. MAA: macroaggregated albumin.

### Statistical Analysis

Values are presented as mean ± standard deviation (SD) or median (25^th^–75^th^ percentiles) for parameters with skewed distribution as mSv. Chi-square test with Yates correction was used to compare categorical data. The unpaired 2-tailed Student’s t-test and the Mann-Whitney test were employed for comparisons. A p-value of<0.05 was considered statistically significant. SPSS (SPSS Inc., Chicago, IL, USA) version 13 was used for all analyses.

## Results

### Study Population

The characteristics of the population at study entry are shown in Table 2. IHD was defined in 63% of patients as history of myocardial infarction, and/or angiographic documentation of significant coronary stenosis and/or chest pain syndrome in spite of angiographically normal coronary arteries (syndrome X or coronary vasospastic angina) or atypical chest pain eventually defined of proven ischemic origin. Non-IHD patients were patients affected by valvular and pericardial disease, cardiomyopathy, non ischemic arrhythmias etc. Traditional risk factors were present in almost 50% of the population. Sixty-eight percent of patients underwent at least one angiographic procedure, and 24% at least one myocardial nuclear medicine scan.

**Table 2:** Clinical characteristics and radiation tests of the study population.

	Overall population (n = 16431)	IHD patients (n = 10350)	Non-IHD patients (n = 6081)	P value
Age (years) mean±SD	62±13	62±12	62±15	0.450
Male sex, n (%)	11,348 (69)	8,125 (78)	3,223 (53)	<0.001
ACS, n (%)		3055(30)		
STEMI/nSTEMI, n(%)		1,668(16)		
Previous MI, n (%)		5,182(50)		
Stable angina, n (%)		2,825(27)		
Variant angina/Syndrome X/etc, n (%)		1,773(17)		
Cardiomyopathy, n (%)	1,823	869 (8)	954(15)	
Other (valvular and pericardial disease, etc), n (%)	4,355(42)	2,009 (19)	2,346(38)	<0.001
History of cigarette smoking, n (%)	7,797(47)	5,770(56)	2,027(33)	<0.001
Hypercholesterolemia, n.(%)	6,768 (41)	4,786 (46)	1,982 (33)	<0.001
Diabetes mellitus, n.(%)	2,885 (17)	2,060 (20)	825 (14)	<0.001
Hypertension, n.(%)	7,446 (45)	4,631 (45)	2,815 (46)	0.054
Coronary angiographies, n (%);	13,604 (82)	10,977(106)	2,627(43)	<0.001
PCIs, n (%)		5,353(52)		<0.001
Number of PCI treated vessels, n (%)		6,838 (66)		
Thallium or Sestamibi scan, n (%)	3,918(24)	3,109 (30)	809 (13)	<.0001
Cumulative estimated effective dose mSv/pt (median, andinter-quartile range)	17(12–38)	27(12–49)	12(4–19)	<.0001

MI: myocardial infarction. PCI: percutaneous coronary intervention. STEMI: ST Elevation Myocardial Infarction**.** nSTEMI: non ST Elevation Myocardial Infarction.

### Radiological Exposures

The most frequent examinations were coronary angiography, percutaneous coronary interventions, and Thallium and Sestamibi scan for myocardial perfusion imaging (Table 2). Males received a significantly higher dose exposure than females (20, 12–42 vs 13, 7–28 mSv, p<0.001).IHD patients received a significant higher dose exposure than non-IHD patients (27, 12–49 vs 12, 4–19, p<0. 0001).

### Radiological Exposure Over Time

**Figure 1:**
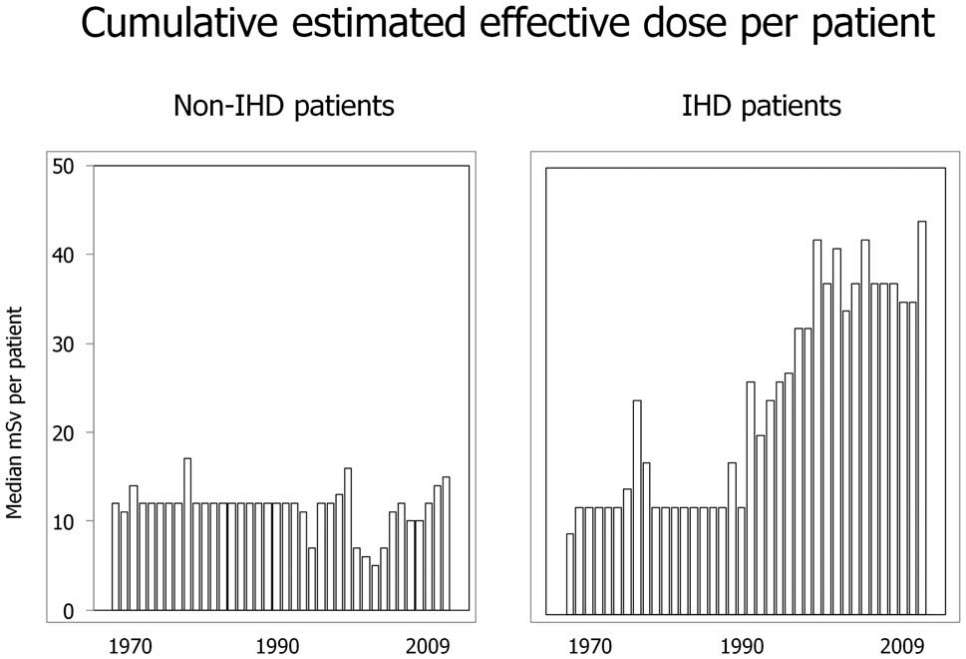
Secular trends of cardiovascular medical radiation exposure over the last 40 years in in-hospital patients admitted for cardiovascular disease. Radiation exposure is expressed as cumulative estimated effective dose/patient. On the left panel the trend of non-ischemic (non-IHD) patients, and on the right the one of ischemic (IHD) patients.

The trends in radiation exposure expressed as cumulative estimated effective dose showed a steady increase in IHD and a flat trend in non-IHD patients, with variation from 1970–74 to 2005–2009 of +155% for IHD (p<0.001) and −1% in non-IHD (NS) (Figure 1). The relative contribution of different imaging techniques was also remodeled over time, with nuclear cardiology (mostly cardiac perfusion scintigraphy) dominating in 1970s (23% of individual exposure) and invasive fluoroscopy (mostly contrast left ventriculography and coronary angiography) and interventional fluoroscopy (mostly percutaneous coronary revascularization) in the last decade (90% of individual exposure).

### Radiological Exposure and Acute Coronary Syndrome

**Figure 2:**
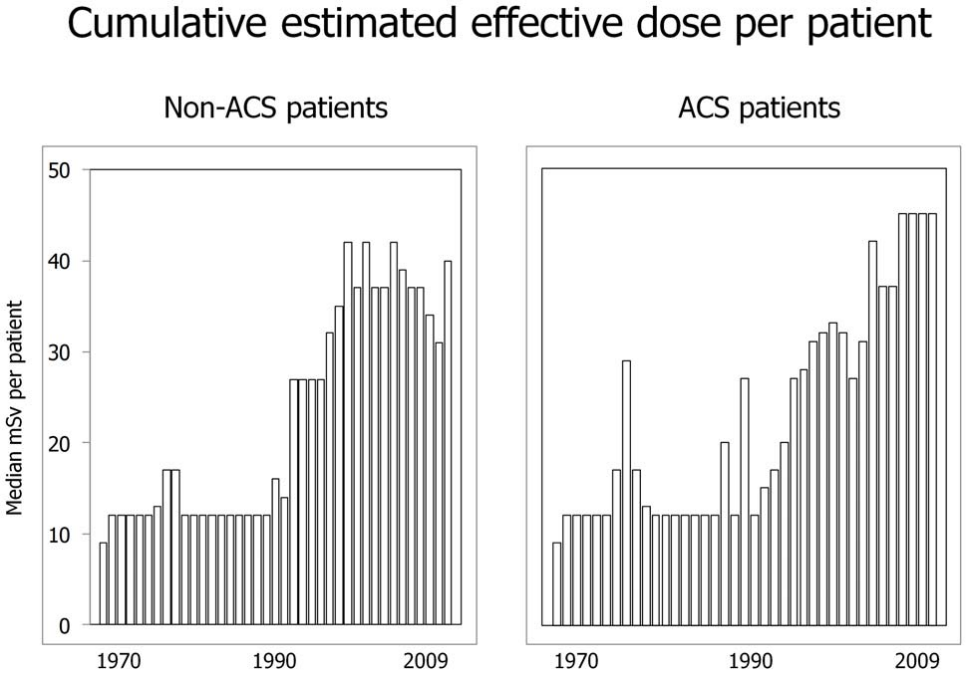
Secular trends of cardiovascular medical radiation exposure over the last 40 years in ischemic (IHD) patients. Radiation exposure is expressed as cumulative estimated effective dose/patient. On the left panel, trend of chronic angina patients (Non-ACS), and on the right trend of acute coronary syndrome (ACS) patients.

In IHD patients a trend toward a progressive increase in cumulative estimated effective dose was observed both in patients hospitalized for ACS and in stable ischemic patients who received a significantly higher dose (23, 12–47 vs 27, 12–51 mSv, p<0.001) (Figure2).

## Discussion

In the last four decades, we observed a steady increase in medical cumulative estimated effective dose in our cardiology patients. This increase was limited to patients with IHD.

### Comparison with Previous Studies

The observed cumulative estimated effective dose trends in our cohort closely mirror overall worldwide trends referring to any kind of medical exposure in citizens of the most affluent countries [17], [18]. This is not surprising, since cardiac imaging is the major source of medical radiation of contemporary patients, accounting for 85% of all nuclear cardiology exposures in the US [18], although this percentage shows substantial geographic heterogeneity [19].The medical radiation exposure profile of the contemporary patient is also consistent with previous evidence suggesting that in adult cardiology patients invasive fluoroscopy accounts for 12% of all examinations, and 48% of the total collective dose [7] of population presented with coronary heart disease. It is also interesting to observe the effects of evolution of medical technology on the temporal trends. In our institute, nuclear medicine started in the 1970s. At the beginning, this innovation led to extensive application for cardiac imaging of left ventricular function (with radionuclide ventriculography) and myocardial perfusion (with Thallium imaging) [20]. The relative decrease of the nuclear medicine contribution in the last decade is due to the development of transthoracic 2-dimensional echocardiography (in the 1980s) and stress echocardiography (1990s), somewhat reducing the applications of myocardial scintigraphy in in-hospital patients [21]. Another source of reduction is the progressive shift of radionuclide tracer from Thallium (100% of all exams in the seventies) to Sestamibi, which has a significantly lower radiation dose [22] and accounted for 95% of all myocardial perfusion imaging examinations in the last 5 years and 100% in the last 3 years. In fact, starting July 2007 our nuclear cardiology officially embargoed Thallium studies for dosimetric reasons.

### Limitations

Our work was retrospective and observational, with the inherent limitations of this study design. However, over the years our data bank has generated very robust information and has intrinsic merit, since for 40 years all in-hospital patients have been followed-up by the coordinated efforts of technicians, statisticians, and physicians, and all diagnostic and therapeutic procedures carefully collected and stored at the time of admission [12], [13].

The radiological history was derived from hospital records, and reference doses, not truly delivered doses, were considered. Reference doses are neither precisely measured nor subject-specific [23] and can vary on the basis of the literature used as reference [24], [16].

The effective dose is a calculated estimate designed to provide a sex-averaged dose for a reference subject in a given exposure situation, not a dose for a specific subject [25]. This calculation relies on assumptions regarding the radiation sensitivity of organs and tissues, imaging techniques and protocols, and, in the case of nuclear imaging, radiopharmaceutical activity, half-life, distribution, and elimination kinetics [25]. Although these assumptions have raised controversy concerning the use of effective dose [26]–[30] it remains the only measure currently available that reflects the overall potential biologic detriment among various types of radiation exposure, which is why we used it as our primary measure of radiation exposure.

There is, in practice, marked dose variability for each exam. The variability is highest for interventional procedures. For example, a coronary stenting procedure is associated with an average effective dose corresponding to 15 mSv, but the individual procedure value may range anywhere between 7 and 57 mSv according to guidelines [16], and even from 3 mSv to 80 mSv in a consecutive series of real patients studied in a high volume cath lab [31], [32]. A similar variation has been associated with noninvasive examinations. For example, examining CT studies performed on adult patients within and across several institutions in the San Francisco Bay area, Smith-Bindman et al. report a mean 13-fold variation in dose indexes between the highest and the lowest dose for each type of study assessed [33]. It is almost certain that the doses of some examinations – especially invasive intervention–have changed substantially over the years, with the evolution of technology, greater experience with the technique, and the diffusion of the culture of radioprotection [34]. Nevertheless, the number of examinations can be considered a reasonably good proxy for radiation exposure, and the cumulative estimated effective dose per patient is likely to give a reliable estimate of the relative irradiation burden within the cohort, rather than precise absolute values, which are likely to be systematically underestimated. As a further limitation, as this data set involves inpatient examinations only, it underestimates total exposure contributed by outpatient testing (especially CT imaging in the current era) and cumulative exposure from subsequent testing over the course of time from both cardiac and non-cardiac diagnostic procedures.

### Clinical Implications

The prognosis of cardiac patients has changed spectacularly over the last 40 years, thanks to reduction in major risk factors and breakthrough advances in cardiac imaging, pharmacological therapy, cardiac surgery, and interventional cardiology [2], [3]. The cardiac patient lives longer and better and this is also witnessed by the relative low rate of cardiac death in contemporary in-hospital population. However, this also means that we should pay greater attention to long-term side effects of our interventions, including imaging procedures using ionizing radiation. These types of imaging procedures have led to improvements in the diagnosis and treatment of numerous cardiological diseases. At the same time, these types of exams expose patients to ionizing radiation, which is a known carcinogen, and a single interventional procedure is associated with increased chromosome aberration in circulating lymphocytes, which are an intermediate endpoint and a long-term predictor of cancer [35]. The contemporary cardiology patient receives a cumulative median effective dose of 60 mSv per head, with 1 out of 4 patients exceeding 100 mSv [7]. This refers to a cumulative, lifetime exposure, with all intra- and out-of-hospital examinations, whereas in the present study only in-hospital exposures are considered. In the United States, high doses (>20–50 mSV/year) from medical imaging procedures were incurred in 2% of the general population [36]. Unfortunately, one-third of our (ionizing and non-ionizing) cardiac imaging exams are partially or totally inappropriate [37], [38]. In our population a significantly higher estimated effective dose was observed in stable ischemic patients in whom the beneficial effect of invasive procedure is less proven [2].We should make every effort to reduce this unnecessary exposure, applying the two main principles of radiation protection: appropriate justification for ordering and performing each procedure, and careful optimization of the radiation dose used during each procedure [39], [40]. In other words, we as cardiologists should be proactive in implementing in our practice the recommendations of the FDA [4]: “Each patient should get the right exam at the right time, with the right radiation dose”.

### Conclusions

A trend to progressive increase in cumulative estimated effective dose is observed in a hospitalized population of cardiovascular patients with IHD, mainly due to extensive use of serial myocardial scintigraphies and invasive fluoroscopy procedures. The increase in radiation exposure is much less marked in patients with non-ischemic heart disease, such as cardiomyopathy, valvular or pericardial disease. This high and rising radiation exposure may further increase in the era of cardiac CT. The growing medical radiation exposure may encourage a more careful justification and optimization policy regarding ionizing imaging in cardiologic patients, often now belonging to a low risk group with benign prognosis and long life expectancy, which should make both the doctors and the patients more conscious of long-term, adverse, potential cancer effects of radiation use.
